# Comparative gut microbiome composition and predicted microbial functions in captive and free-range yaks (*Bos grunniens*)

**DOI:** 10.14202/vetworld.2026.864-876

**Published:** 2026-02-28

**Authors:** Bingbing Ye, Ruilan Liu, Rongqing Li, Mohd Rohaizad Md Roduan, Wan Syaidatul Aqma Wan Mohd Noor, Fareed Sairi

**Affiliations:** 1 Key Laboratory of Ecological Protection and Characteristic Industry Cultivation in Hengduan Mountain Area, Sichuan Minzu College of Sichuan Provincial Department of Education, Ganzi Prefecture, Kangding, 626001, Sichuan, China; 2 Department of Biological Sciences & Biotechnology, Faculty of Science & Technology, Universiti Kebangsaan Malaysia, Bangi 43600, Selangor, Malaysia; 3 UKM Culture Collection (UKMCC), Faculty of Science & Technology, Universiti Kebangsaan Malaysia, Bangi 43600, Selangor, Malaysia

**Keywords:** Yak (*Bos grunniens*), Free-range vs. captive yaks, Gut microbiomes, metabarcoding, functional prediction

## Abstract

**Background and Aim:**

The gut microbiota is essential for nutrient digestion, immune function, and environmental adaptation in ruminants, particularly high-altitude species like yaks (*Bos grunniens*). Different husbandry practices (captive vs. free-range) can potentially alter the microbial communities and affect the yak health. However, comparative data on how these systems affect yak gut microbiomes remain limited, with most studies focusing on taxonomy rather than functional implications. This study aimed to compare gut microbiome composition, diversity, and predicted functional profiles between captive (CY) and free-range (FY) yaks using a 16S rRNA gene metabarcoding approach.

**Materials and Methods:**

Fecal samples were collected from healthy ~2-year-old yaks (n=5 CY, n=5 FY) in Litang County, Ganzi Prefecture, Sichuan, China, during summer. DNA was extracted, and the V4 region of the *16S rRNA* gene was sequenced on Illumina NovaSeq 6000. Bioinformatic analyses included quality filtering, Operational taxonomic units (OTU) clustering (97% similarity), taxonomic annotation (SILVA database), α- and β-diversity analysis. The microbial function was predicted using PICRUSt2 (KEGG pathways), BugBase (community phenotypes), and FAPROTAX (ecological functions). Statistical comparisan used Welch’s t-tests, Wilcoxon rank-sum tests, principal coordinates analysis (PCoA), and Analysis of similarities (ANOSIM) with significance set at p < 0.05.

**Results:**

α-Diversity indices (e.g., Shannon p = 0.5476) showed no significant differences between CY and FY. However, β-diversity revealed distinct community structures (PCoA: PC1 30.52%, PC2 12.25%; ANOSIM R = 0.976, *p* = 0.007), with FY samples more homogeneous. At the genus level, CY were enriched in *Ruminococcaceae* bacterium UCG-005, *Streptococcus*, *Escherichia*-*Shigella*, *Treponema*, *Christensenellaceae* R-7, and *Clostridium sensu stricto* 1 (many fermentative or potentially opportunistic). FY showed higher abundances of *Bacillus*, *Arthrobacter*, *Rhodococcus*, *Candidatus Saccharimonas*, *Prevotellaceae* UCG-001, and *Paenibacillus*. Predicted functions indicated FY had greater capacities for carbohydrate/amino acid metabolism, DNA repair, fatty acid biosynthesis, and vitamin B pathways, while CY favored fermentation and reductive acetogenesis. BugBase highlighted higher anaerobic phenotypes in CY.

**Conclusion:**

Husbandry practices profoundly influence yak gut microbiome structure and inferred metabolic potential, with free-range systems promoting, homogeneous communities suited to natural high-fiber diets while captive systems promotes fermentative and opportunistic shifts. These microbiome differences suggest opportunities for probiotic interventions to enhance yak health, productivity, and sustainability in high-altitude pastoral systems. Future metagenomic and metabolomic validation is needed.

## INTRODUCTION

The yak (*Bos grunniens*) is a rare ruminant breed that lives on high-altitude plateaus above 3,000 m. They are well adapted to the harsh environments of their habitats, including low temperatures, high humidity, high atmospheric pressure, intense UV radiation, and limited food availability [1, 2]. These unique adaptations allow yaks to thrive in extreme environments, making them indispensable to livelihoods in regions such as the Ganzi Prefecture, where they provide meat, milk, and transportation [3]. As of 2021, Ganzi Prefecture has more than 1.7 million yaks (11.5% of China’s national total and 41.07% of the provincial herd), underscoring their importance to the local economy [4]. Therefore, optimizing yak health and productivity is essential for the sustainability of high-altitude pastoral systems. Given the central role of the gut microbiota in ruminant health, feed efficiency, and environmental resilience, understanding how different husbandry practices shape the yak gut microbiome can inform targeted breeding and management strategies to enhance their productivity and sustainability [5].

The gut microbiota is important for ruminant digestion, nutrient utilization, immune regulation, and adaptation to environmental stress. It contributes by breaking down complex plant fibers and proteins into key nutrients for the host [6]. However, microbial composition may be affected by various factors, such as age, season, nutrition, and environment, with diet being the most influential factor [7]. Different husbandry practices (free-range and captive breeding) may also alter gut microbiota composition, thereby affecting the host’s metabolic growth and development [8].

Comparative studies have demonstrated that yak gut microbiome diversity varies with both geographic region and feeding system. Geographic differences have been linked to marked shifts in microbial composition, with *Bacteroidota*, *Firmicutes*, and *Actinobacteriota* being the dominant phyla in certain regions [9]. Captive yaks have a significantly higher Chao1 index than their free-ranging counterparts, likely due to differences in dietary composition. However, captive feeding may also promote the enrichment of non-fiber and hemicellulose-degrading taxa, which can alter the fermentation pathways and reduce nutrient utilization efficiency [10].

Metagenomic analyses of ruminant gut microbiomes have also revealed the presence of pathogenic bacteria and antimicrobial resistance genes, which pose risks to both animal health and food safety. Pathogens, such as *Escherichia*-*Shigella* and *Acinetobacter*, have been associated with gastrointestinal disorders and antibiotic resistance [11]. Collectively, these findings suggest that husbandry practices and feeding strategies not only modulate microbial diversity but also reshape functional capacities, with significant implications for animal productivity and public health.

Despite these insights, current findings on the gut microbiome of *B. grunniens* remain inconsistent across geographic regions and feeding conditions, making it challenging to isolate the specific effects of husbandry practices alone [9, 10, 12]. Most existing studies have focused primarily on taxonomic composition and α-diversity indices such as Chao1, with limited attention to β-diversity patterns, community homogeneity (as revealed by principal coordinates analysis (PCoA) and Analysis of similarities (ANOSIM)), or the functional implications of microbial shifts under CY versus FY systems. Moreover, predictive functional profiling using tools such as PICRUSt2, BugBase, and FAPROTAX has rarely been applied to yaks, leaving a critical gap in understanding how husbandry-driven microbial changes may influence nutrient metabolism, fermentation pathways, vitamin biosynthesis, and overall host adaptability in high-altitude environments. This lack of integrated taxonomic and functional data hinders the development of microbiome-informed management strategies tailored to *B. grunniens* in pastoral systems.

Therefore, the present study aimed to compare the gut microbial composition, diversity patterns (including α- and β-diversity), and predicted functional profiles between CY and FY in the same geographic region using high-throughput 16S rRNA gene sequencing. By combining taxonomic profiling with functional predictions via PICRUSt2 (KEGG pathways), BugBase (community phenotypes), and FAPROTAX (ecological functions), we sought to elucidate how husbandry practices shape the yak gut microbiome and to provide foundational insights for optimizing diet, health, and productivity in high-altitude yak production systems.

## MATERIALS AND METHODS

### Ethical approval

All procedures in this study were conducted in accordance with the ethical guidelines of Sichuan Minzu College and the relevant national standards for animal welfare in China (including GB/T 35892-2018: Laboratory Animal – Guideline for Ethical Review of Animal Experiments). The study was purely observational and non-invasive, involving only the collection of fresh fecal samples from the ground or spontaneously defecated material without any physical restraint, handling, capture, anesthesia, or intervention that could cause harm, pain, distress, or behavioral alteration to the *B. grunniens*). No experimental treatments, invasive procedures, or manipulations were performed.

Given the non-invasive nature of fecal collection (which did not involve direct animal contact beyond observation from a distance and posed no risk to animal welfare), formal ethical review or approval by an Institutional Animal Care and Use Committee or equivalent ethics committee was not required under Sichuan Minzu College guidelines and applicable Chinese regulations for such low-risk, non-experimental livestock studies.

Informed consent was obtained from all yak owners/herders prior to sample collection. Consent was documented verbally and in writing (in Tibetan and Mandarin as appropriate), with full explanation of the study’s purpose, methods, non-invasive approach, potential benefits for yak health and husbandry, data confidentiality, and voluntary participation (Supplement 3 for consent templates and records). No compensation was provided, and owners retained the right to withdraw consent at any time.

This approach complies with international best practices for non-invasive research on the microbiomes of wildlife and livestock, including principles from the ARRIVE 2.0 guidelines (for reporting animal research) and recommendations from the World Organisation for Animal Health Terrestrial Animal Health Code on minimizing animal disturbance. All sampling was performed by trained personnel using sterile techniques to prevent contamination and environmental impact. The study adhered to the 3Rs principle (Replacement, Reduction, Refinement) by using non-invasive methods and limiting sample size to the minimum required for scientific validity.

### Sample collection

Fecal samples were collected from yaks raised in Li Tang County, Ganzi Tibetan Autonomous Prefecture, Sichuan, China, in June (summer season). During sampling, the local climate was characterized by cool alpine summer conditions (typical temperature range: 8°C–18°C) with abundant natural forage growth.

Captive yaks were sampled at 100°17′9.600′′E, 29°58′55.200′′N (average elevation: 3908.0 m; Li Tang Yak Eco-industry Yak Park), and free-range yaks were sampled at 100°21′3.600′′E, 29°48′43.200′′N (average elevation: 3831.5 m). All animals originated from a single herd within their respective systems. Free-range yaks co-grazed on a shared alpine pasture, dominated by natural forage species typical of the region (*Kobresia* spp., alpine grasses, and native forbs), and used the same natural water source. Captive yaks were housed in a single farm unit at the Litang County Zangyuan Animal Husbandry under uniform management.

The captive diet consisted primarily of green hay (the main roughage source) supplemented with feed made from corn and soybean meal, reflecting the typical nutrient composition of local captive yak production. Although detailed chemical composition (DM, CP, NDF, ADF, and minerals) was not available from the farm records, all animals within the captive system received the same feed batch.

Because the animals belonged to local herder-owned production systems rather than controlled experimental herds, detailed metadata, including body weight, body condition score, sex, vaccination history, deworming records, parasite screening, and recent antibiotic use, were not recorded by the owners. However, all selected animals were approximately 2 years old, belonged to the same herd, and showed no visible signs of illness, diarrhea, or injury at the time of sampling. Only animals that were judged healthy by the herders were included.

A total of 10 fresh fecal samples were collected (n = 5 captive; n = 5 free-range). Sampling occurred between 9:00 and 10:00 a.m. The outer fecal surface of each sample was removed using a sterile cotton swab, and a new sterile swab was used to collect fecal material from the inner portion to minimize environmental contamination. The samples were placed immediately into sterile 2-mL tubes. The samples were transported to the laboratory on dry ice and stored at −80°C until DNA extraction. All samples were simultaneously processed to avoid batch effects.

### DNA extraction and polymerase chain reaction amplification

Total genomic DNA was extracted from fecal samples using the HiPure Stool DNA Mini Kit (Magen Company, Guangzhou, China) according to the manufacturer’s protocol. DNA integrity was examined by agarose gel electrophoresis, and the concentrations were quantified using a Qubit 3.0 fluorometer (Thermo Fisher Scientific, Waltham, MA, USA).

The bacterial *16S rRNA* gene was amplified targeting the V4 hypervariable region using primers 515F (5′-GTGYCAGCMGCCGCGGGTAA-3′) and 806R (5′-GGACTACNVGGGGTWTCTAAT-3′), a widely validated primer pair for gut microbiota profiling owing to its broad coverage and robust taxonomic resolution. The PCR reaction mixture (25 μL) contained template DNA, 2× PCR master mix (Vazyme Biotech, Nanjing, China), and primers at the recommended concentrations. The thermal cycling program included an initial denaturation at 95°C for 5 min; 30 cycles of 94°C for 30 s, 57°C for 40 s, and 72°C for 60 s; followed by a final extension at 72°C for 10 min. To monitor potential contamination, each PCR batch included a negative control (nuclease-free water). PCR products were verified by agarose gel electrophoresis, purified, ligated to sample-specific barcodes, and quantified using Qubit prior to sequencing. Sequencing libraries were constructed and sequenced on an Illumina NovaSeq 6000 platform (Illumina, San Diego, CA, USA) using paired-end 2 × 250 bp chemistry [13].

### Bioinformatics analysis

#### Preprocessing of the raw reads

The quality control of raw paired-end reads was performed using FASTP (version 0.18.0). Reads were removed if they contained ≥10% ambiguous bases (N), showed adapter contamination, or had more than 50% of bases with a Phred quality score ≤20. Paired-end reads passing quality filters were merged into tags using FLASH v1.2.11 with a minimum overlap of 10 bp and a maximum mismatch rate of 2% [14]. Raw tags were further filtered by truncating sequences at the first position where three consecutive bases had Q ≤3, and tags were discarded if the remaining high-quality region accounted for <75% of the total length. Clean high-quality tags were retained for downstream analysis [15].

#### Operational taxonomic units (OTU) clustering and taxonomic assignment

OTUs were clustered at 97% similarity using the UPARSE algorithm in USEARCH v11.0.667, and the most abundant sequence within each OTU was selected as the representative sequence [16]. UCHIME was used to identify and remove chimeric sequences. The RDP Classifier v2.2 was used to perform taxonomic annotation against the SILVA *16S rRNA* gene reference database (version 138.1), with an 80% confidence threshold [17].

#### Analysis of diversity and functional prediction

Alpha-diversity indices, including Chao1, ACE, Shannon, Simpson, and Good’s coverage, were calculated in R (version 4.3.1) using standard equations referenced from Mothur. Rarefaction curves and rank–abundance curves were generated using the ggplot2 package (version 3.2.1) in R to evaluate sequencing depth and community evenness. Beta-diversity analysis was conducted based on unweighted UniFrac distance to assess phylogeny-based differences in microbial community composition, and PCoA was performed using the vegan package (version 2.5.6) in R [18]. For functional prediction, PICRUSt2 (version 2.5.3) was used to predict microbial metabolic functions based on the KEGG database. To improve prediction reliability, samples with Nearest Sequenced Taxon Index values greater than 2 were excluded. Microbial phenotypes were predicted using BugBase (version 1.0), and FAPROTAX (version 1.2.4) was used to annotate ecological functions.

### Statistical analysis

All statistical analyses were performed in R and the Omicsmart online platform. Alpha-diversity indices and taxonomic relative abundances were compared between groups using Welch’s t-tests (two-tailed), with statistical significance set at p < 0.05. Multivariate analyses were performed using PCoA based on distance matrices, and ANOSIM with 999 permutations was applied to evaluate differences in community structure between groups. Spearman correlation coefficients were calculated for network analysis to construct co-occurrence networks. Correlations with p < 0.05 were retained, and the top 50 strongest correlation pairs were visualized using the Omicsmart platform’s dynamic interactive data analysis tools. When the data did not meet the normality assumptions, the non-parametric Wilcoxon rank-sum tests were applied as appropriate.

## RESULTS

### Data collection and OTU analysis

**Figure 1:**
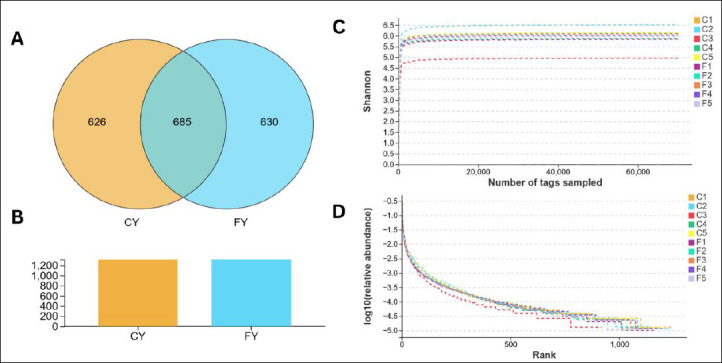
OTU distribution and sequencing depth assessment. (A) Venn diagram. (B) OTU histogram. (C) Shannon rarefaction curve. (D) Species abundance curve.

Amplicon sequencing on 10 collected yak fecal samples yielded 1,288,404 raw sequences (CY = 650,001, FY = 638,403) (Table 1). After quality assessment and data filtering, a total of 1,027,471 (CY = 502,805; FY = 524,666) qualified sequences were obtained from the 10 samples. These valid sequences were clustered into 3,617 operational taxonomic units (OTUs) based on 97% nucleotide sequence similarity. For downstream analyses, OTUs with a mean tag count ≥1 within a group were retained. As shown in the Venn diagram (Figure 1A), both groups shared a large core microbiota (685 OTUs), whereas the CY and FY groups shared 826 and 630 OTUs, respectively (Figure 1B). The coverage of the dominant taxa was adequate, as indicated by the rapid rise of Shannon rarefaction curves and the attainment of stable plateaus by 10,000 reads across all samples. Most libraries showed similar Shannon diversity (≈5.8–6.3), although one captive sample (C3) remained consistently lower (Figure 1C). Furthermore, rank–abundance plots (log10 scale) indicated community structures with long rare-taxa tails that were broadly similar, except for C3. The latter displayed a steeper decline with a shorter tail, consistent with reduced richness and evenness relative to the other samples (Figure 1D).

**Table 1:** Sequencing statistics for captive (CY) and free-range (FY) yak fecal samples

Sample name	Raw reads	Clean reads	Raw tags	Clean tags	Chimera	Effective tags	Effective ratio (%)
C1	132020	131913	129866	128748	27135	101613	76.97
C2	130072	129951	128034	127120	27953	99167	76.24
C3	122743	122640	120565	119739	23186	96553	78.66
C4	133853	133731	131781	130723	26730	103993	77.69
C5	131313	131207	129233	128330	26851	101479	77.28
F1	131884	131736	129916	129417	16123	113294	85.90
F2	130964	130686	128619	127768	23492	104276	79.62
F3	123133	122965	121047	120309	22486	97823	79.44
F4	132275	132101	130059	129269	24058	105211	79.54
F5	120147	119837	118061	117565	13503	104062	86.61

### Analysis of α-diversity between captive and free-range yaks

**Figure 2:**
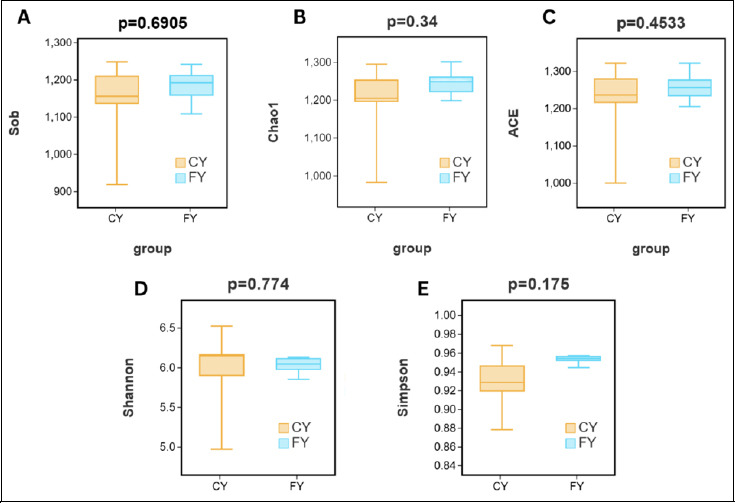
Box plots of statistical tests for the diversity of intestinal microbial communities in captive and free-ranging yaks. (A) The Sob index of bacterial diversity. (B) Chao1 index of bacterial diversity. (C) ACE index of bacterial diversity. (D) Shannon index of bacterial diversity. (E) Simpson index of bacterial diversity.

The α-diversity of intestinal flora in yaks with different husbandry practices indicates no significant difference between the FY and CY groups, despite the FY group consistently exhibiting higher diversity and species richness. As depicted in Figure 2, Wilcoxon rank-sum tests for observed richness (Sob), Chao1, ACE, Shannon, and Simpson showed no significant differences between CY and FY (Shannon p = 0.5476; ACE p = 0.8413; Chao1 p = 0.4206; Simpson p = 0.2222; Sob p = 0.6905; all p > 0.05). Thus, no group-level differences in richness or evenness were detected at the chosen rarefaction depth, implying that husbandry or feeding style did not significantly alter the overall α-diversity of yak gut microbiota.

### Analysis of β-diversity between captive and free-range yaks

**Figure 3:**
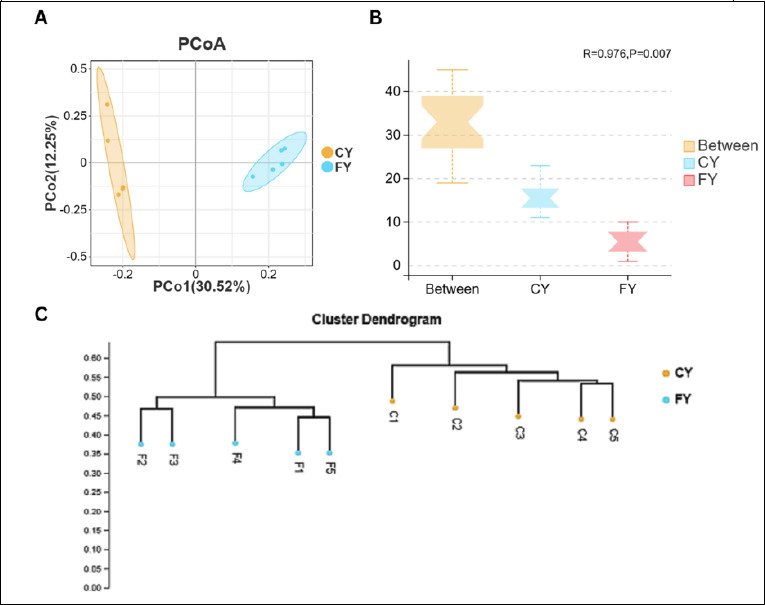
(A) Principal coordinate analysis (PCoA) based on unweighted UniFrac distance showing clear separation between captive (CY) and free-range (FY) yaks. (B) Analysis of similarities (ANOSIM) confirms that between-group dissimilarities exceeded within-group dissimilarities (R = 0.976, p = 0.007). (C) UPGMA hierarchical clustering based on unweighted UniFrac distance, grouping samples according to husbandry system.

While α-diversity reflects within-sample richness and evenness, β-diversity analysis was used to assess how microbial community composition differed between CY and FY yaks. As shown in Figure 3A, PCoA based on unweighted UniFrac distance revealed a clear separation of gut microbial communities between the CY and FY groups, with PC1 (30.52%) and PC2 (12.25%) together explaining 42.77% of the total variance. Notably, the FY samples formed a more compact cluster, indicating lower within-group dispersion (i.e., greater community homogeneity) than the CY samples.

Analysis of similarities (ANOSIM) demonstrated that between-group dissimilarities were significantly greater than within-group dissimilarities (R = 0.976, p = 0.007) (Figure 3B). The Unweighted Pair Group Method with Arithmetic Mean hierarchical clustering tree based on unweighted UniFrac distance further showed that samples clustered primarily according to the husbandry system, with CY and FY forming distinct branches (Figure 3C). Collectively, these results indicate that although the overall within-sample diversity did not differ significantly between groups, husbandry practice was associated with pronounced differences in microbial community composition, with free-range yaks exhibiting more homogeneous gut microbiota than captive yaks.

### Composition of the gut microbiota of captive vs. free-range yaks across taxonomic ranks

**Figure 4:**
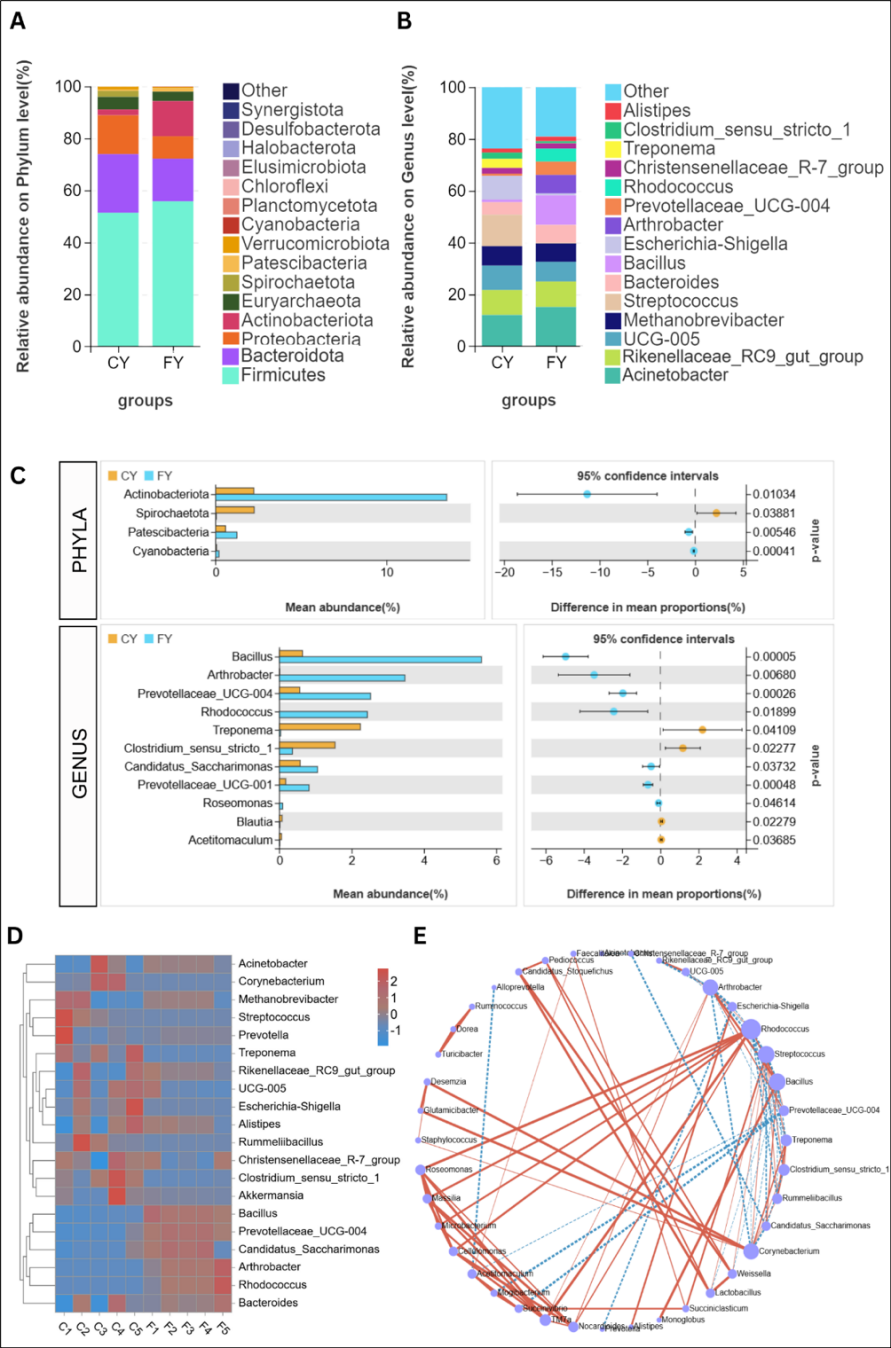
The gut microbiome structure of captive (CY) and free-range (FY) yaks. Overall community composition per group according to stacked bars of the top 15 phyla (A) and 15 genera (B). (C) Comparison of phyla and genera for captive (CY) and free-range (FY) according to Welch’s t-test. (D) Z-score heatmap of individuals in each group against genera with hierarchical clustering. (E) Co-occurrence network of key genera; node size ∝ connectivity (degree). Edges denote significant pairwise associations (p < 0.05); solid red = positive co-occurrence; dashed blue = negative co-occurrence.

Amplicon-based metagenomic profiling of fecal samples from free-range (FY; F1–F5) and captive (CY; C1–C5) yaks revealed 20 phyla, 31 classes, 81 orders, 127 families, and 233 genera. At the phylum level, *Firmicutes* (CY: 51.25%, FY: 55.69%) and *Bacteroidota* (CY: 22.49%, FY: 16.36%) dominated the communities across all individuals (Figure 4A). Beyond this core, husbandry-linked differences were observed at both the phylum and genus levels (Figure 4A–B). FY showed higher *Actinobacteriota*, *Patescibacteria*, and *Cyanobacteria*, whereas CY exhibited higher *Proteobacteria*, *Euryarchaeota*, *Spirochaetota*, *Verrucomicrobiota*, and *Planctomycetota* (respective means: *Proteobacteria*—CY 14.87%, FY 8.56%; *Actinobacteriota*—CY 2.24%, FY 13.53%; *Euryarchaeota*—CY 4.81%, FY 3.58%; *Spirochaetota*—CY 2.26%, FY 0.03%; *Patescibacteria*—CY 0.57%, FY 1.24%; *Verrucomicrobiota*—CY 1.10%, FY 0.51%; *Cyanobacteria*—CY 0.04%, FY 0.19%; *Planctomycetota*—CY 0.04%, FY 0.01%). However, only *Actinobacteriota*, *Patescibacteria*, *Cyanobacteria*, and *Spirochaetota* differed significantly between groups (Figure 4C), with *Spirochaetota* being notably enriched in CY.

At the genus level (Figure 4B), several communities were clearly separated by husbandry practice. CY were enriched with *Streptococcus* (7.72% vs. 0.11% in FY), *Escherichia*–*Shigella* (5.78% vs. 0.34%), *Rikenellaceae_ RC9_ gut_group* (6.12% vs. 4.80%), *Ruminococcaceae* bacterium UCG-005 (6.03% vs. 3.71%), and *Methanobrevibacter* (4.79% vs. 3.44%). *Bacillus* (5.59% vs. 0.64% in CY), *Arthrobacter* (3.47% vs. 0.001%), and *Prevotellaceae_UCG-004* (2.52% vs. 0.56%) were higher in free-range yaks (FY), whereas *Acinetobacter* (7.70% CY; 7.40% FY) and *Bacteroides* (3.10% CY; 3.37% FY) were similar between groups. *Treponema* and *Clostridium_sensu_stricto_1* were significantly higher in CY than FY (p < 0.05) (Figure 4C). FY were characterized by greater abundances of *Bacillus* (5.59% vs. 0.64% in CY), *Arthrobacter* (3.47% vs. 0.001%), and *Prevotellaceae_UCG-004* (2.52% vs. 0.56%), with *Rhodococcus* (p < 0.01), *Candidatus Saccharimonas* (p < 0.05) and *Prevotellaceae_UCG-001* (p < 0.01) also significantly enriched in FY.

The genus level differences between CY and FY were mirrored at the individual-sample level, as depicted in the genus heatmap (Figure 4D). All FY (F1–F5) formed a coherent cluster characterized by higher signals for *Bacillus*, *Arthrobacter*, *Prevotellaceae_UCG-004*, *Rhodococcus*, and *Candidatus Saccharimonas*, whereas CY (C1–C5) co-clustered with elevated *Streptococcus*, *Escherichia*–*Shigella*, *Treponema*, *Corynebacterium*, *Clostridium_ sensu_stricto_1*, *Methanobrevibacter*, *Ruminococcaceae* UCG-005, and *Rikenellaceae_ RC9_ gut_ group*. Genera with similar group mean, such as *Acinetobacter* and *Bacteroides*, showed mixed intensities across both clusters. Consistently, these patterns were supported by β-diversity analysis based on unweighted UniFrac PCoA, which separated FY and CY with minimal overlap, consistent with the patterns observed in Figure 3. The heatmap and β-diversity results demonstrate that FY have a more homogeneous community, whereas captive yaks have a more heterogeneous community.

The co-occurrence network (Figure 4E) demonstrated two opposing modules that aligned with the previous results. An FY module centered on *Bacillus*, *Arthrobacter*, *Prevotellaceae_UCG-004*, and *Rhodococcus* showed dense positive connectivity (e.g., *Bacillus*–*Prevotellaceae_UCG-004* r = 0.88; *Arthrobacter*–*Rhodococcus* r = 0.89), while exhibiting negative associations with CY taxa (e.g., *Streptococcus*–*Bacillus* r = −0.96; *Streptococcus*–*Prevotellaceae_UCG-004* r = −0.84). Conversely, a CY module includes *Streptococcus*, *Clostridium_sensu_stricto_1*, *Treponema*, *Corynebacterium*, *Ruminococcaceae* UCG-005, *Rikenellaceae_RC9_gut_group*, and *Escherichia*–*Shigella*, which are positively interconnected (e.g., *Streptococcus*–*Clostridium_s.s._1* r = 0.79; *Treponema*–*Clostridium_s.s._1* r = 0.77; UCG-005–*Escherichia*–*Shigella* r = 0.88) and negatively linked to the FY module (e.g., *Bacillus*–*Clostridium_s.s._1* r = −0.76; *Arthrobacter*–*Treponema* r = −0.84). Hubs by degree include *Rhodococcus* (15 edges), *Bacillus* (12 edges), *Streptococcus* (12 edges), *Arthrobacter* (11 edges), and *Corynebacterium* (11 edges), reinforcing the FY vs. CY polarity seen in Figs. 4B–D and the β-diversity separation (Supplement 1).

Together, the compositional, statistical, clustering, and network results show that although both groups share a *Firmicutes*–*Bacteroidota* core, the yak gut microbiome was structured into two distinct community states by different husbandry practices. While FY harbors a more homogeneous community and forms a dense and positively co-occurring module, CY exhibits a more heterogeneous assemblage, which positively co-occurs and shows negative associations with the FY module. However, these results should be interpreted with caution because they represent exploratory indications of potential co-occurrence patterns rather than evidence of direct biological interactions.

### Functional prediction

**Figure 5:**
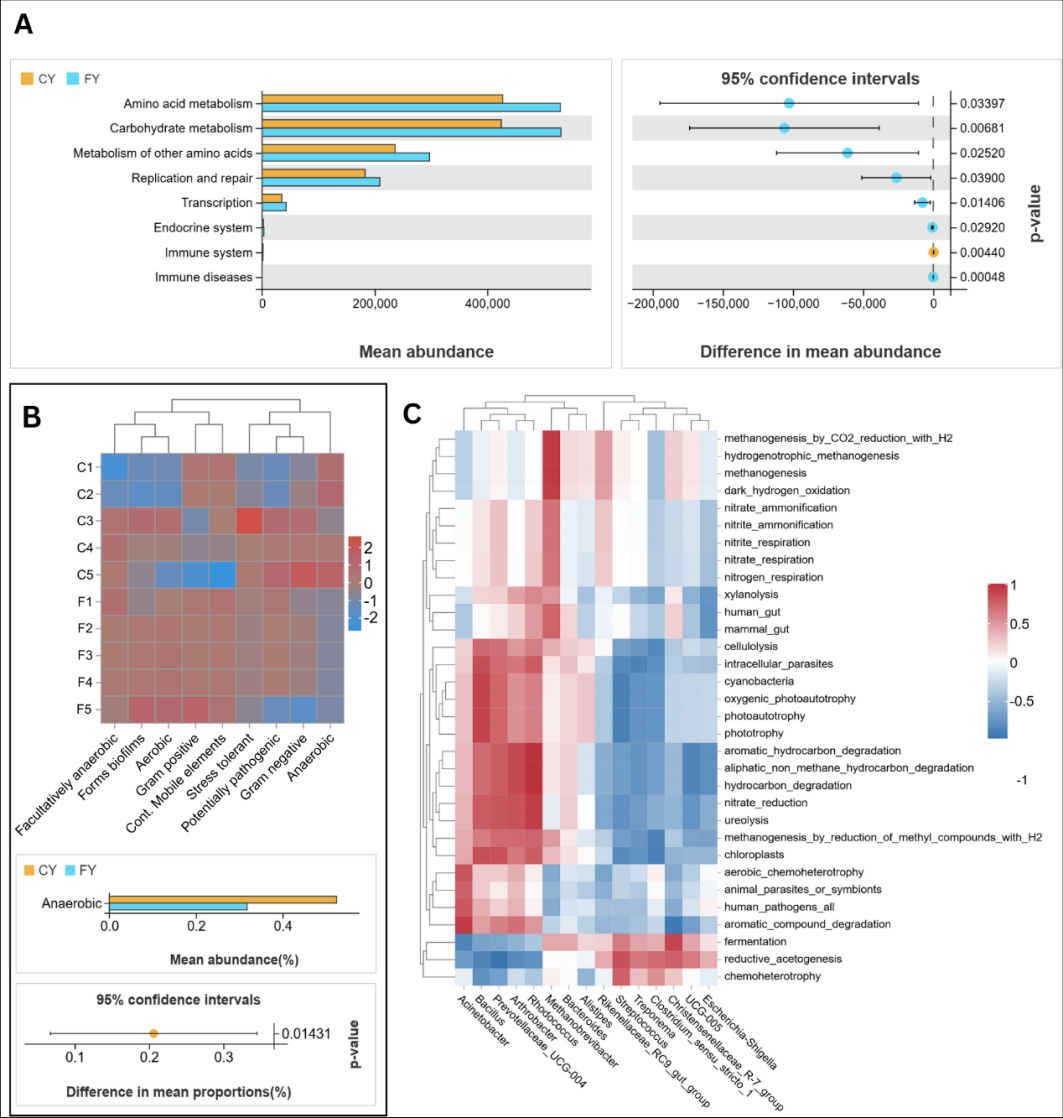
(A) PICRUSt2 KEGG predicted metabolic pathway abundance in group means (CY vs. FY), mean difference with 95% CI. KEGG L2 labels reflect bacterial gene annotations only. (B) BugBase phenotype structure across samples (top: clustered heatmap); bottom: statistical group difference (p-value < 0.05). (C) Spearman correlations between FAPROTAX ecological functions and the top 15 genera. p ≤ 0.001, p ≤ 0.01, p ≤ 0.05. Rows grouped by functions; columns by the top 15 genera.

It should be noted that all functional profiles reported here are predictions inferred from *16S rRNA* gene data rather than direct measurements, and therefore should be interpreted as putative and hypothesis-generating. Taxonomically, both groups shared a *Firmicutes*–*Bacteroidota* core, but FY was enriched in fibrolytic/saccharolytic taxa (e.g., *Bacillus*, *Arthrobacter*, *Prevotellaceae* UCGs, *Rhodococcus*, *Candidatus Saccharimonas*), whereas CY harbored *Streptococcus*, *Escherichia*–*Shigella*, *Ruminococcaceae* UCG-005, *Rikenellaceae_RC9_gut_group*, *Treponema*, and *Clostridium_sensu_stricto_1*. Consistent with this composition, PICRUSt2 predicted 169 KEGG level 3 pathways, spanning Metabolism (~70%), Genetic Information Processing (~11%), Cellular Processes (~6%), disease-related pathways (labeled as “Human Diseases” in the KEGG hierarchy; ~5%), Environmental Information Processing (~4%) and Organismal Systems (~3%). FY showed greater predicted capacities for carbohydrate and amino acid metabolism and higher replication and repair (Figure 5A).

At KEGG level 3 (Supplement 2), the FY enrichment was driven by the central-carbon and fiber-driven routes. The metabolic pathways involved include glycolysis/gluconeogenesis, pyruvate metabolism, the citrate (TCA) cycle, the pentose-phosphate pathway, amino-sugar and nucleotide-sugar metabolism, glyoxylate/dicarboxylate metabolism, and propanoate/butanoate metabolism, as well as broad amino acid pathways (alanine/aspartate/ glutamate; glycine/serine/threonine; cysteine/methionine; arginine/proline; histidine; tryptophan) and cofactor/ vitamin biosynthesis (pantothenate–CoA, folate one-carbon pool, lipoic acid). FY also showed higher fatty acid biosynthesis, including unsaturated fatty acids. However, the CY group displayed selective increases in carotenoid biosynthesis, sesquiterpenoid and triterpenoid biosynthesis, type II polyketide backbone biosynthesis, and nitrotoluene degradation. Notably, most of the other xenobiotic degradation routes (bisphenol, styrene, atrazine, and polycyclic aromatic hydrocarbons) were higher in FY. The community phenotype from BugBase also supports this metabolic split, as the anaerobic phenotype was significantly higher in CY (Welch’s t, p = 0.01431), whereas other phenotypes showed no groupwise differences (Figure 5B).

In addition to metabolic pathway prediction, the ecological functions of the gut microbiota were predicted using FAPROTAX and linked to the top 15 genera using a dynamic correlation heatmap (Figure 5C). Chemoheterotrophy, fermentation, and reductive acetogenesis were positively correlated with CY-enriched taxa (*Treponema*, *Streptococcus*, *Clostridium_sensu_stricto_1*, and *Christensenellaceae_R-7*) and negatively correlated with FY-favored genera (*Bacillus*, *Prevotella*, *Arthrobacter*, and *Rhodococcus*). The latter also positively correlated with hydrocarbon degradation, aromatic hydrocarbon degradation, and aliphatic-non-methyl hydrocarbon degradation, whereas *Bacillus* exhibited only mild links to general chemoheterotrophy. On the other hand, taxa present in both groups, such as *Acinetobacter* and *Methanobrevibacter*, were shown to correlate positively with aromatic compound degradation and methanogenesis/H_2_ oxidation/nitrogen metabolism, respectively.

## DISCUSSION

### Community structure and β-diversity patterns

The effects of different husbandry practices on the diversity and structure of yak gut microbiota were investigated using high-throughput sequencing of the *16S rRNA* V4 region from fecal samples. Overall, the results indicate that while captive (CY) and FY maintain comparable α-diversity, their gut microbial communities differ in composition and predicted functional profiles under the two husbandry systems. The α-diversity analysis revealed no significant differences between the CY and FY groups across the Sob, Chao1, ACE, Shannon, and Simpson indices (p > 0.05), indicating that both husbandry systems support microbial communities with similar richness and diversity. A slight, non-significant tendency toward higher richness- and diversity-related indices in FY yaks is consistent with reports that high-fiber grazing diets can support complex microbial communities in ruminants [19, 20], although both CY and FY groups appear to maintain relatively resilient intestinal microbiota [2, 21]. In contrast, β-diversity based on PCoA showed clear separation between the two groups, with FY samples clustering more tightly than CY samples. This pattern indicates a more homogeneous community structure among FY yaks and greater between-animal variation in CY yaks [22], although the present data cannot determine the underlying mechanisms. Recent studies have demonstrated that yaks rely heavily on their gut microbiota to cope with the Qinghai–Tibet Plateau’s harsh conditions [23], and the distinct community structures observed here may reflect different microbial configurations under free-range and captive management.

### Taxonomic shifts between captive and free-range yak populations

Firmicutes and Bacteroidetes were the most dominant phyla in both groups at the phylum level, consistent with previous observations in ruminants [24]. In this study, the total abundance of Firmicutes (CY: 51.25%, FY: 55.69%) and Bacteroidetes (CY: 22.49%, FY: 16.36%) did not differ significantly between groups. Firmicutes in the gastrointestinal tract can effectively degrade cellulose and hemicellulose, contributing to energy extraction from fibrous plant material [25], whereas Bacteroidetes participate in the degradation of non-fibrous complex polysaccharides and in maintaining gut balance [26]. The Firmicutes-to-Bacteroidetes ratio has been proposed as an indicator of fiber degradation capacity in ruminants [27–29]. However, in the present study, the differences in this ratio between CY and FY were modest and should be interpreted cautiously. At the genus level, differential abundance analysis revealed distinct shifts in dominant microbial taxa between the CY and FY groups, highlighting the influence of feeding regime on gut microbiota composition. Treponema and *Clostridium_sensu_stricto_1* were significantly enriched in the CY group. *Treponema* participates in the degradation of plant-derived polysaccharides, particularly lignocellulose [30]. *Clostridium_sensu_stricto_1* includes species with strong fermentative capacity that produce short-chain fatty acids (SCFAs), but some members may also have pathogenic potential [31].

The enrichment of pathogenic indicators, such as *Escherichia*–*Shigella*, in captive yaks warrants attention, and similar patterns have been observed in other captive ruminants where altered community structure may increase susceptibility to pathogenic colonization [32, 33]. These observations suggest that certain management practices in captivity could be associated with a higher relative abundance of potentially opportunistic taxa, although this study did not assess the direct impacts on health. In contrast, the FY group exhibited higher relative abundances of genera with reported probiotic or metabolic benefits. *Bacillus* is a well-known probiotic that produces carbohydrate-active enzymes that facilitate the breakdown of complex polysaccharides and enhance nutrient use [34]. Recent studies have confirmed the probiotic potential of *Bacillus* strains isolated from Tibetan yaks, demonstrating their ability to improve digestive efficiency and immune function [29]. Some FY-enriched genera, such as Arthrobacter, participate in nitrogen transformations, including nitrite reduction [35], which may contribute to gut nitrogen metabolism. *Rhodococcus*, although better known as a soil bacterium, exhibits considerable metabolic versatility, including the degradation of diverse organic compounds [36], and its presence in FY samples may indicate additional metabolic capacities, but its specific role in the yak intestine remains to be clarified. *Candidatus_Saccharimonas* has been associated with carbohydrate metabolism and immune modulation, possibly contributing to intestinal homeostasis [37]. Furthermore, Prevotellaceae_UCG-001 is a fiber-degrading genus commonly enriched in herbivorous animals, and its abundance in FY yaks likely reflects the high-fiber diet derived from natural grazing [38]. These taxonomic patterns are consistent with diet-related differences in microbial substrates between captive and free-range systems.

### Predicted functional implications

The functional profiles of the gut microbiota were predicted using PICRUSt2, BugBase, and FAPROTAX. The taxonomic and predicted functional differences observed between CY and FY yaks suggest that free-range management may favor gut microbial communities with greater potential for fiber degradation and diverse metabolic capacities under high-altitude grazing conditions, whereas captive feeding may be associated with distinct fermentative and potentially opportunistic taxa. Inferred functional pathways suggested that FY communities may have a higher potential for fiber-related carbohydrate metabolism and central-carbon processing, including xylan/cellulose use and glycolysis–TCA-related routes [39–41]. The predicted enrichment of propanoate and butanoate metabolism in FY yaks is consistent with a possible increase in VFA production, which is important for ruminant energy supply [42]. At the same time, predicted nitrogen transformation functions and amino acid biosynthesis pathways may indicate capacity for ammonia assimilation and microbial protein formation [43, 44]. FY yaks also showed predicted enrichment of B-vitamin/cofactor biosynthesis and fatty acid–related pathways, which could contribute to vitamin supply and lipid precursors [45].

However, all of these functional profiles are based on *16S rRNA* gene–based inferences rather than direct metagenomic, metatranscriptomic, or metabolomic analysis. Therefore, these predictions should be viewed as putative and hypothesis-generating evidence of metabolic activity [46]. Further work integrating rumen microbiota, host physiological parameters, and metabolite profiles will be required to confirm these interpretations and to clarify their relevance for yak health and high-altitude adaptation.

### Study limitations

This study has several limitations. First, the sample size was small (n = 10) due to the constraints of field sampling in remote pastoral regions, which limited the statistical power of the differential abundance and functional inference analyses. Second, PICRUSt2, BugBase, and FAPROTAX provide inference-based functional profiles rather than direct measurements; therefore, metagenomic and metabolomic validation is required. Third, key metadata (e.g., sex, body weight, physiological status, parasite burden, antibiotic history, and detailed dietary intake) were unavailable in herder-managed livestock and may have confounded the observed associations, despite sampling animals of similar age from a single herd within each system. Fourth, the cross-sectional design precludes inference about temporal dynamics or causality, and only fecal samples were analyzed, which may not fully reflect the rumen microbiota. Finally, fermentation metabolites (e.g., SCFAs) were not measured, limiting the link between microbial shifts and host metabolism. Overall, the findings should be viewed as preliminary and hypothesis-generating.

## CONCLUSION

This study demonstrated that husbandry practices significantly shape the gut microbiome of yaks (*B. grunniens*), even though overall α-diversity remained comparable between captive (CY) and free-range (FY) groups. No significant differences were detected in α-diversity indices, indicating similar richness and evenness within samples. However, β-diversity analysis revealed clear separation between CY and FY communities, with FY samples forming a tighter, more homogeneous cluster compared to the greater dispersion observed in CY. Taxonomically, both groups shared a *Firmicutes*–*Bacteroidota* core, but distinct shifts occurred at lower ranks: CY were enriched in *Streptococcus*, *Escherichia*–*Shigella*, *Treponema*, *Clostridium sensu stricto 1*, *Ruminococcaceae* bacterium UCG-005, and *Rikenellaceae RC9 gut group*, while FY showed higher abundances of *Bacillus*, *Arthrobacter*, *Rhodococcus*, *Candidatus Saccharimonas*, *Prevotellaceae UCG-001*, and *Prevotellaceae UCG-004*. Functional predictions via PICRUSt2, BugBase, and FAPROTAX indicated that FY communities possessed greater potential for carbohydrate and amino acid metabolism, fatty acid biosynthesis, vitamin B pathways, and DNA replication/repair, whereas CY favored fermentation, reductive acetogenesis, and anaerobic phenotypes. These findings suggest opportunities for targeted dietary interventions (e.g., fiber supplementation, probiotic strains such as *Bacillus* isolates) or microbiome-informed management strategies to improve feed efficiency, health, and productivity in captive yak herds. However, the conclusions are associative and limited by the small sample size and reliance on *16S rRNA* gene–based functional predictions. Future studies incorporating shotgun metagenomics, metabolomics, controlled feeding trials, and larger cohorts with more complete metadata are needed to validate the predicted functions and clarify the microbial mechanisms underlying yak adaptation and health in different management systems.

## DATA AVAILABILITY

The original sequencing data generated in this study have been deposited in the National Center for Biotechnology Information Sequence Read Archive (SRA). The data are available under the National Center for Biotechnology Information BioProject accession number PRJNA1380723.

## AUTHORS’ CONTRIBUTIONS

YB: Conceptualization, methodology, and writing—original draft preparation. LR: Conceptualization, methodology, data curation, and writing—original draft preparation. FS: Methodology and writing—review and editing. MR: Writing—review and editing. WS: Writing—review and editing. All authors have read and approved the final version of the manuscript.
